# Patient–Prosthesis Mismatch in Contemporary Small-Size Mechanical Prostheses Does Not Impact Survival at 10 Years

**DOI:** 10.3390/jcdd9020048

**Published:** 2022-01-31

**Authors:** Horea Feier, Mihaela Mocan, Andrei Grigorescu, Lucian Falnita, Marian Gaspar, Constantin-Tudor Luca

**Affiliations:** 1 Department of Cardiology, University of Medicine and Pharmacy “Victor Babes” Timisoara, 300041 Timisoara, Romania; horea.feier@umft.ro (H.F.); ae.grigorescu@gmail.com (A.G.); mariangaspar24@yahoo.ro (M.G.); costiluca67@yahoo.ro (C.-T.L.); 2 Research Center of the Institute for Cardiovascular Diseases, 300310 Timisoara, Romania; lfalnita@gmail.com; 3 Department of Cardiology, University of Medicine and Pharmacy Iuliu Hatieganu Cluj-Napoca, 400012 Cluj-Napoca, Romania

**Keywords:** aortic valve, mechanical valve, patient–prosthesis mismatch, propensity matching

## Abstract

Background: The effect of PPM in mechanical prostheses on long-term survival is not well-established. Methods: Patients who received a 21 mm or smaller aortic valve between 2000 and 2011 were retrospectively analyzed (*n* = 416). Propensity matching was used in order to account for baseline differences in patient subgroups (PPM vs. no PPM; severe PPM vs. no severe PPM). Results: Five- and ten-year survival was 78 ± 3.52% and 64.51 ± 4.51% in patients with PPM, versus 83.3 ± 3.12% and 69.37 ± 4.36% in patients without (*p* = 0.28) when analyzed at 10.39 ± 5.25 years after the primary procedure. Independent risk factors for impaired survival, after matching, were age, serum creatinine, and severe pulmonary hypertension. Five- and ten-year survival in patients with severe PPM was 73.34 ± 6.01% and 61.76 ± 8.17%, respectively, versus 74.72 ± 5.68% and 67.50 ± 7.09% in those without (*p* = 0.49), at 8.82 ± 5.17 years after SAVR. Age was the only independent variable that influenced long-term survival when severe PPM was added to the model. Conclusions: PPM or severe PPM does not impact long-term survival up to 10 years in mechanical valve recipients when matching for preoperative variables.

## 1. Introduction

Aortic stenosis is the most prevalent valve disease in the adult population, reaching 9.8% in 80-year-old patients [1]. Surgical aortic valve replacement (SAVR) has been the only available treatment for symptomatic as well as asymptomatic patients with an aortic valve area <1 cm^2^, a reduced left ventricular ejection fraction (LVEF), or undergoing surgery for another indication [2,3]. Mechanical prostheses are still the substitute of choice for young patients (<55 years), but the availability of percutaneous valve-in-valve techniques (TAVR) has challenged the role of these substitutes in patients aged 55–65, particularly those with a high surgical risk, making the use of mechanical substitutes questionable.

Patient–prosthesis mismatch (PPM), defined as an effective orifice area (EOA) of the valve substitute that is too small for the body surface area of the patient, has been identified by Rahimtoola [4], investigated extensively, and found to be responsible for reduced long-term survival [5,6]. These results have been disputed, however, in cohorts that used modern valve substitutes [7,8,9]. This discussion gained increasing relevance, as percutaneous prostheses have better hemodynamics than stented valves.

The small aortic root has been defined in surgical series as an aortic annulus accepting an aortic prosthesis smaller than or equal to 21 mm in size [10,11]. The prevalence of PPM is increased in this population, so that its influence on long-term survival may be stronger.

We sought to investigate whether PPM limits long-term survival in contemporary mechanical valve recipients.

## 2. Materials and Methods

### 2.1. Data Collection

This report represents a comparative retrospective single-center study. Patient data were collected during treatment using standardized forms to record demographic and clinical characteristics as well as procedural and follow-up data. Follow-up was obtained using medical records, patient interviews, and National Health Register data. The study protocol was approved by the Ethical Board of our institution. Due to the retrospective nature of the study, written consent was waived.

### 2.2. Patient Population

We reviewed the hospital records of patients operated on in our department from 1 January 2000 to 31 December 2011. We included in the study all patients that received a mechanical valve sized 21 mm or smaller in the aortic position, irrespective of the underlying pathology, and found 416 consecutive subjects that satisfied these criteria. While most patients underwent surgery for isolated aortic stenosis, associated procedures included coronary artery bypass and mitral valve or ascending aortic replacements. 

The effective orifice area (EOA) of the prostheses that were implanted were taken from manufacturers’ data sheets, as previously reported and validated [12,13,14]. 

Within these cohorts of mechanical valve recipients, we compared the long-term results of those with PPM versus those without PPM, evaluated at ≥10 years after the initial SAVR. 

### 2.3. Definitions

PPM was defined as an EOAi ≤ 0.85 cm^2^/m^2^. Severe PPM was considered to be present if EOAi ≤ 0.65 cm^2^/m^2^. Early mortality was death occurring within 30 days of operation, in-hospital or not. Severe pulmonary hypertension (PHT) was defined as a systolic pulmonary artery pressure superior to 60 mmHg. Peripheral vascular disease (PVD) was defined as a >50% stenosis in the carotid, subclavian, or peripheral arteries. Coronary artery disease (CAD) was considered to be present when a major epicardial artery presented a stenosis >50%. Chronic obstructive pulmonary disease (COPD) was defined by spirometry as a VEMS lower than 80% of the normalized value under inhalatory medication. Renal function was evaluated using the Cockroft–Gault formula, and an estimated glomerular filtration rate less than 60 mL/min/m^2^ was considered to define renal dysfunction. Body surface area (BSA) was computed using Mosteller’s formula. Body mass index (BMI) was derived by the standard formula. Cerebrovascular disease was defined by the history of a cerebrovascular accident. 

### 2.4. Propensity Matching

**Figure 1:**
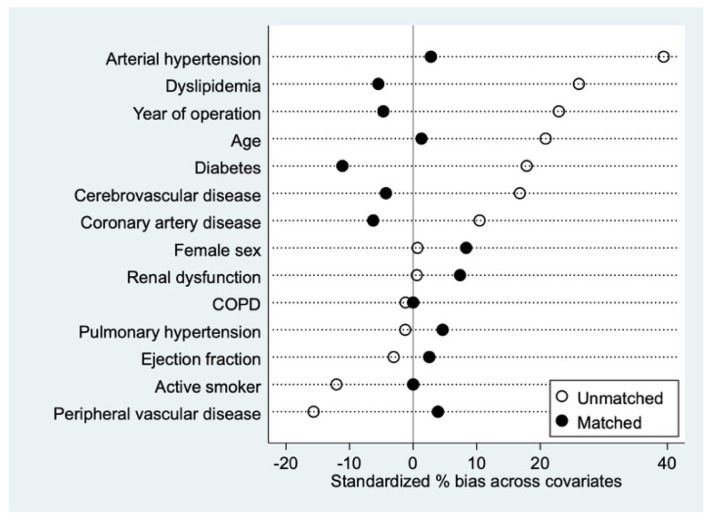
Bias before and after propensity matching.

Patients who had PPM/severe PPM were matched 1:1 with a 0.2 standard deviation caliper, using matching without replacement by means of the “psmatch2” statistical package in Stata, to those not exhibiting PPM/severe PPM. Matching covariates were chosen that could alter long-term survival and were the following: year of operation, age, sex, ejection fraction, severe pulmonary hypertension, coronary artery disease, dyslipidemia, arterial hypertension, active smoking, chronic obstructive pulmonary dysfunction, renal dysfunction, diabetes, peripheral vascular disease, and cerebrovascular disease. Covariate balance after matching was assessed by computing the standardized % bias, with less than 10% considered acceptable (Figure 1).

The baseline characteristics of our patients as well as the matched cohort are presented in Table 1. 

### 2.5. Objectives

The primary endpoint was overall survival. Patients were confirmed as dead by telephone interview of the relatives, and the exact date of death was found in the National Health Register. Follow-up was 100% complete. The secondary outcome of interest was a composite endpoint of death or reoperation for a prothesis-related issue. These were performed for major paravalvular leak, endocarditis, PPM, or valve thrombosis. Patients that were subsequently reoperated for another issue, such as aortic surgery, coronary artery bypass, or mitral valve, were not censored in the survival analysis. 

### 2.6. Statistical Analysis

Normal distribution was assessed using the Shapiro–Wilk test. The primary outcome of interest was survival, while the secondary outcome was survival without reoperation. Univariate analysis was performed using t-tests and Mann–Whitney or chi-squared tests. Time-to-event analysis was performed using log-rank or Kaplan–Meier estimates. Cox proportional hazard regression was performed for the primary and secondary outcomes, with data reported as hazard ratios and 95% confidence intervals. The proportional hazard assumptions were tested using Schoenfeld residuals analysis. Linearized rates of death or reoperation were calculated as being the incidence of the event divided by total patient-years of follow-up and reported for the matched population. The average treatment effect on the treated was estimated by using the “stteffects” command from the matched sample by inverse-probability weighting, with female sex, BSA, and BMI as covariates that would predict PPM allocation, while year of operation, age, sex, ejection fraction, severe pulmonary hypertension, coronary artery disease, dyslipidemia, arterial hypertension, active smoking, chronic obstructive pulmonary disease, renal dysfunction, diabetes, peripheral vascular disease, and cerebrovascular disease were the covariates that would influence long-term survival. Multivariate survival analysis was performed on the matched population using covariates that would influence survival. Statistical testing was performed using Stata 16.1 (StataCorp LLC, College Station, TX, USA). A *p* value less than 0.05 was deemed to assess statistical significance. 

## 3. Results

Among 416 unmatched patients, there were 100 aortic valve replacements with prostheses size 19 or smaller (five patients with Medtronic OpenPivot size 18), while 316 received a 21 mm prosthesis. The types of prostheses used in the unmatched sample, as well as their EOAs, are presented in Table 2. 

### 3.1. PPM 

Matching yielded two groups of 147 patients that received a 21 mm or smaller implant who either exhibited or did not exhibit PPM (Table 1). There was a single patient in which annulus enlargement was performed in the matched sample; all others received a 21 mm or smaller mechanical aortic valve implant. We implanted a mechanical prosthesis whenever we encountered an extremely small aortic annulus, even in patients >65 years, if 19 mm biologic substitutes were not available. Cross-clamp and bypass times were similar (Table 3). Thirteen patients had a postoperative length-of-stay >15 days. Five patients who required a longer postoperative stay had a 19 mm valve implanted, while eight had a 21 mm. 

Early mortality (<30 days) was 5.78% and was higher in patients with PPM (8.16% vs. 3.4%), but this did not reach statical significance (*p* = 0.08). The predicted mortality, by the Euroscore 2 risk score, of these patients was 3.67 ± 3.39 (*p* < 0.01). 

**Figure 2:**
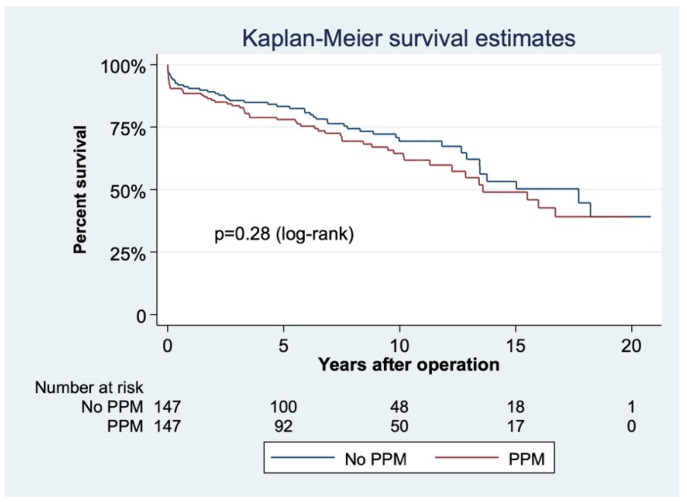
Cumulative survival in the matched cohort according to the presence of patient–prosthesis mismatch (EOAi ≤ 0.85 cm^2^/m^2^).

The matched cohort had 2287 patient-years of follow-up (7.78 ± 5.26 years). Median follow-up in this cohort was 7.41 years (3.27, 11.14). Time from the primary procedure was 9.92 years (6.79, 12.91). Five- and ten-year survival were 78 ± 3.52% and 64.51 ± 4.51% in patients with PPM versus 83.3 ± 3.12% and 69.37 ± 4.36% in patients without it (*p* = 0.28, Figure 2) when matching for preoperative variables.

**Figure 3:**
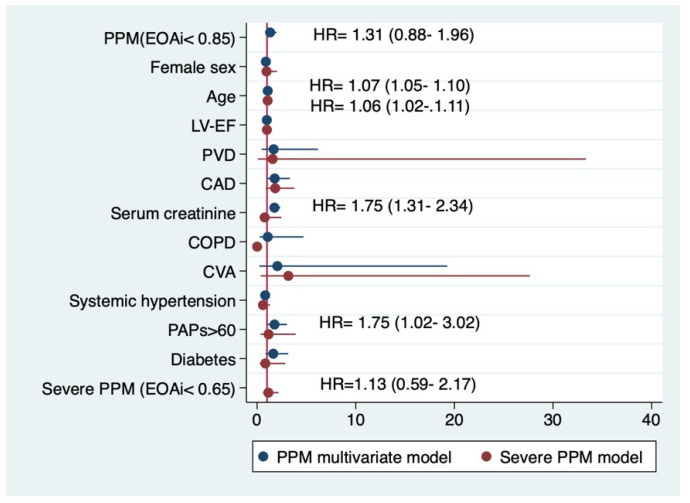
Independent risk factors for impaired long-term survival. The numbers represent the hazard ratio (HR) and the 95% CI. LVEF = left ventricular ejection fraction; PVD = peripheral vascular disease or carotid stenosis >50%; CAD = a stenosis of at least 50% in a major epicardial coronary vessel; COPD = chronic obstructive pulmonary disease diagnosed by spirometry and/or under inhalatory medication; CVA = previous cerebrovascular accident. PAPs = systolic pulmonary artery pressure.

Independent risk factors for mortality within the matched population included older age, serum creatinine, and preoperative severe pulmonary hypertension (Figure 3). The Schoenfeld residuals test returned a probability of 0.81 for the proportionality assumption to be met by the included variables. Death incidence rate was 0.039 (95% CI 0.029–0.052) for subjects with no PPM and 0.049 (95% CI 0.037–0.064) for those exhibiting PPM after the primary procedure (*p* = 0.25).

The average effect of PPM on the recipients were estimated using the “stteffects” command from the matched sample by inverse-probability weighting. There were no differences in survival in the matched sample, in patients with or without PPM (*p* = 0.76, Table 4).

There were two reoperations in our matched sample for valve-related issues, both of them in 19 mm valve recipients who exhibited PPM, increased gradients across their aortic valves, and pannus formation at 7 and 11 years after the primary procedure, respectively. The linearized rate of reoperation was 0.08% per patient-year overall.

**Figure 4:**
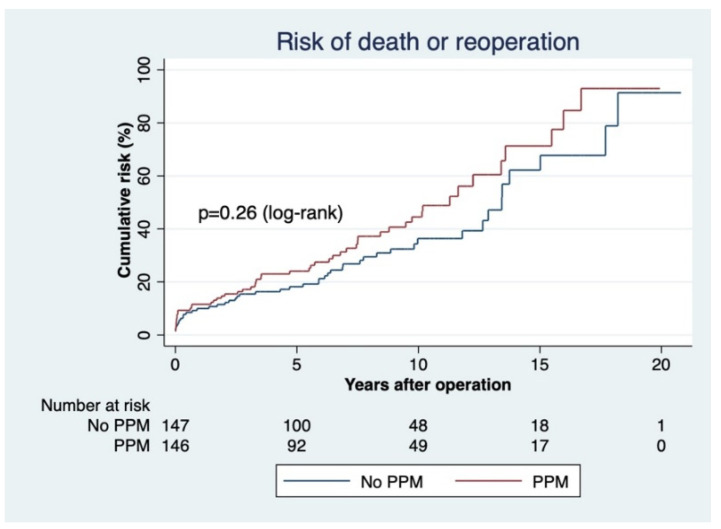
Composite risk of death or reoperation in the matched cohort according to the presence of patient–prosthesis mismatch (EOAi ≤ 0.85 cm^2^/m^2^).

The secondary endpoint of survival without death or reoperation was met at 10 years by 69.37 ± 4.36% of patients without PPM and by 63.90 ± 4.57% of those with PPM in the matched population (*p* = 0.26, Figure 4).

### 3.2. Severe PPM

**Figure 5:**
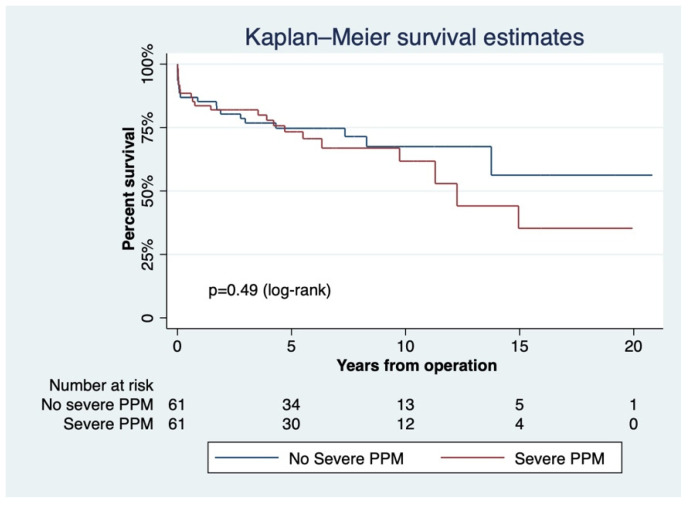
The effect of severe patient–prosthesis mismatch (EOAi ≤ 0.65 cm^2^/m^2^) on long-term survival.

Propensity matching yielded two groups of 61 subjects that either exhibited severe PPM or not. Early mortality was 9.84% and was similar between patients with severe PPM (8.20%) or without (11.48%, *p* = 0.54). The predicted mortality was 1.92 ± 1.72 (*p* < 0.01). The matched cohort had 745 patient-years of follow-up (6.10 ± 4.86 years). Time from the primary procedure was 7.17 years (4.93, 11.28). Long term-survival in patients with severe PPM was 73.34 ± 6.01% and 61.76 ± 8.17% at 5 and 10 years, respectively, versus 74.72 ± 5.68% and 67.50 ± 7.09% in those without (*p* = 0.49, Figure 5).

Independent risk factors for mortality within the matched population included only older age when severe PPM was added to the multivariate model (Figure 3). The Schoenfeld residuals test returned a probability of 0.83 for the proportionality assumption to be met by the model. The incidence of death was 0.080 (95% CI 0.049–0.131) for subjects with severe PPM and 0.040 (95% CI 0.032–0.050) for those without (*p* = 0.01). The average treatment effect of severe PPM on the postoperative survival of patients was not found to significantly alter the long-term outcome in the matched cohort (Table 5).

## 4. Discussion

The incidence of aortic stenosis has increased due to longer life expectancy afforded by advances in healthcare, making it the most prevalent valve pathology. Surgical aortic valve replacement (SAVR) has been the treatment of choice for aortic stenosis, but the development of TAVR has challenged this [15,16,17]. 

Rahimtoola was the first to introduce the notion of PPM into clinical practice [4]. Pibarot and Dumesnil have extensively investigated this issue [18,19,20,21] and reported a multitude of conclusions, which supported the deleterious effect of PPM on short-term mortality [19], particularly in patients with pre-existing left-ventricular dysfunction. This group had also claimed that PPM would impact long-term survival [22]. Patients in this study had a mean follow-up of 4.8 ± 3.4 years, and 78% of those received a stented or stentless bioprosthesis. Fallon and colleagues, working on the data available through the STS Cardiac Surgery Adult Database, in a large cohort of over 59.000 valve implants, of which 96% were with biologic substitutes, underscored the long-term risk posed by PPM for readmissions for heart failure and reoperation for redo AVR due to prosthetic valve degeneration [23]. On the other hand, Ruel and colleagues, working on a cohort of evenly implanted bioprosthetic (46%) and mechanical valve (54%) recipients have demonstrated that reduced long-term survival due to PPM is present only in patients with pre-existing left-ventricular systolic dysfunction (LVEF < 50%) but not in those with a normal ejection fraction [24]. Other authors have also failed to find an association between PPM and long-term survival up to 20 years, even in very small aortic substitute recipients [25,26]. Functional recovery after SAVR does not seem to be affected by PPM [27], a fact also acknowledged by Pibarot and Dumesnil [18] but disputed by other authors [28,29]. LV mass regression, however, is universally found to be less in patients with PPM after SAVR [24,30].

The effect of PPM on long-term survival seems to be more apparent in studies that include predominantly bioprosthetic valve recipients. There are few studies to date, to the best of our knowledge, that investigate the long-term consequences of PPM on mechanical valve recipients. The Mayo Clinic group, working on small size (19 mm and 21 mm) mechanical St. Jude prostheses recipients, found that moderate-term survival, at a median of 4.8 years, was reduced in patients with severe PPM but not in those with moderate PPM [31]. Their definition of severe PPM (EOAi ≤ 0.6 cm^2^/m^2^) was more restrictive than the one used in this study (EOAi ≤ 0.65 cm^2^/m^2^) and, indeed, in most others on this topic. These authors did not use any form of propensity score matching to account for potential pre-existing confounders. Kohsaka and colleagues, working on a cohort of 469 consecutive SAVR patients who received mechanical aortic valve substitutes, demonstrated a borderline effect of PPM (*p* = 0.05), in a propensity score-adjusted multivariate model, at a median follow-up of 7.9 years [32]. 

It is worth noting that the definition proposed by Rahimtoola for defining moderate (EOAi = 0.65–0.85 cm^2^/m2) and severe (EOAi ≤ 0.65 cm^2^/m^2^) mismatch, which was used for most of the studies on this topic, has been modified by the VARC-2 investigators for obese patients (BMI ≥ 30 kg/m^2)^) to 0.6–0.9 cm^2^/m^2^ for a moderate mismatch and <0.6 cm^2^/m^2^ for severe mismatch [33]. 

To account for potential pre-existing confounders that might influence long-term survival, we used propensity matching to balance the two groups of patients (PPM vs. no PPM; severe PPM vs. no severe PPM). Preoperative variables used for matching were female sex, age, year of surgery, ejection fraction, coronary artery disease, peripheral vascular disease, cerebrovascular disease, renal dysfunction, COPD, diabetes, smoking, systemic hypertension, dyslipidemia, and severe pulmonary hypertension. When accounting for these preoperative factors, postoperative survival—at least up to 10 years of follow-up—was not found to be inferior in subjects who exhibited either moderate or severe PPM after the primary valve replacement procedure with contemporary mechanical substitutes. 

An explanation for the discrepancy between survival in mechanical and biologic valve substitutes might be the fact that valve calcification in bioprosthetic valves seems to be accelerated in the presence of PPM [34], which, in turn, would worsen the transprosthetic gradients in these patients and increase the deleterious effects of PPM on the valve cusps in a positive feedback loop. This mechanism obviously does not work in mechanical valve substitutes, which could explain the difference in survival, with worse long-term results in patients with bioprosthetic aortic valve substitutes who exhibit PPM [23]. On the other hand, reduced LV hypertrophy and mass regression in patients with PPM [35] is still one factor that may contribute to potentially worse survival, but this has not been consistently demonstrated, at least at follow-ups of 10 years or less.

Usage of multidetector computer tomography for assessing the EOA [36] may offer a more accurate appreciation of valve orifice area with less examiner bias. At least in the case of TAVR valves, computer tomography downgrades the frequency of PPM compared to transthoracic cardiac echography [37], and this may further explain the lack of association of echography-derived PPM to the long-term outcome.

### Study Limitations

The main limitation of the present study is that follow-up echocardiographic data were not consistently available. If recent ultrasound data had been available, they would have allowed actual in vivo assessment of EOA; however, examiner variation would have limited comparisons. At the same time, we were not able to ascertain the exact cause of death of the patients in our cohort, which is a major shortcoming of retrospective studies, as this would have allowed us to take into consideration only deaths due to cardiovascular factors. Restricting the study to valves by a single manufacturer, as done by the Mayo group [31], or a single valve type would give homogenous information about these substitutes but could not necessarily be extrapolated to all mechanical prostheses. Another limitation is the inability to draw definitive conclusions about the influence of PPM on outcomes beyond 10 years. In our sample, there was a trend for patients with PPM to fare worse 10 years after the primary procedure, but to investigate this would require a longer follow-up and a higher number of patients. In the present sample, nevertheless, at 10 years after the procedure, PPM had no influence on survival in mechanical aortic prosthesis recipients.

## 5. Conclusions

Moderate to severe patient–prosthesis mismatch in contemporary mechanical valves in the aortic position did not influence long-term all-cause mortality. If present, differences might appear only beyond 10 years after the primary procedure. Matching for other preoperative variables that influence survival mitigates the effect of patient–prosthesis mismatch on survival. There was a trend for severe PPM to reduce survival beyond 10 years of follow-up, which needs further investigation in a larger cohort.

## Figures and Tables

**Table 1:** Baseline characteristics of the study cohort.

Variable	All Patients			Matched Cohort		
	PPM (*n* = 242)	No PPM (*n* = 174)	*p* Value	PPM (*n* = 147)	No PPM (*n* = 147)	*p* Value
	*n* (%)	*n* (%)		*n* (%)	*n* (%)	
Age	60.24 ± 11.04	57.81 ± 13.15	0.04	58.82 ± 12.19	58.66 ± 12.12	0.90
Female sex	147 (60.74)	107 (61.49)	0.87	94 (63.95)	88 (59.86)	0.47
Aortic stenosis	222 (91.74)	163 (93.68)	0.45	135 (91.84)	137 (93.20)	0.65
Systemic hypertension	162 (66.94)	81 (46.55)	<0.01	76 (51.70)	74 (50.34)	0.81
Severe pulmonary hypertension	21 (8.68)	16 (9.20)	0.85	17 (11.56)	15 (10.20)	0.70
Diabetes	47 (19.42)	23 (13.22)	0.09	12 (8.16)	18 (12.24)	0.24
CAD ^1^	33 (13.64)	18 (10.34)	0.31	12 (8.16)	15 (10.20)	0.54
COPD ^2^	6 (2.48)	3 (1.72)	0.60	3 (2.04)	3 (2.04)	1.00
Dyslipidemia	131 (54.13)	70 (40.23)	<0.01	58 (39.46)	62 (42.18)	0.63
PVD ^3^	6 (2.48)	7 (4.02)	0.37	4 (2.72)	3 (2.04)	0.70
Cerebrovascular disease	9 (3.72)	3 (1.72)	0.23	1 (0.68)	2 (1.36)	0.56
LVEF ^4^	51.82 ± 9.28	52.14 ± 11.37	0.75	52.21 ± 10.19	51.94 ± 11.34	0.83
Moderate mitral regurgitation	52 (21.49)	30 (17.24)	0.28	27 (18.37)	29 (19.73)	0.76
BSA ^5^	1.84 ± 0.19	1.72 ± 0.20	<0.01	1.82 ± 0.20	1.73 ± 0.20	<0.01
Mean aortic gradient	52.51 ± 21.02	55.29 ± 20.71	0.19	53 ± 21.90	55.49 ± 20.60	0.32
BMI ^6^	27.92 ± 4.83	25.36 ± 4.62	<0.01	27.76 ± 4.76	25.58 ± 4.77	<0.01

^1^ CAD = a stenosis of at least 50% in a major epicardial coronary vessel; ^2^ COPD = chronic obstructive pulmonary disease diagnosed by spirometry and/or under inhalatory medication; ^3^ PVD = peripheral vascular disease or carotid stenosis >50%; ^4^ LVEF = left ventricular ejection fraction; ^5^ BSA = body surface area measured by Mosteller’s formula; ^6^ BMI = body mass index.

**Table 2:** Types of prostheses implanted in the unmatched sample.

Producer	Valve Type	Unmatched Cohort				Total
		<19 mm (*n*)	EOA (cm^2^)	21 mm (*n*)	EOA (cm^2^)	
St. Jude	Masters	26	1 ± 0.2	87	1.4 ± 0.2	113
	Regent	5	1.6 ± 0.4	25	2 ± 0.7	30
	HP	1	1.3 ± 0.2	0		1
Carbomedics	TopHat	25	1	96	1.4	121
	Orbis	14	1	29	1.5	43
	Standard	9	1 ± 0.4	6	1.5 ± 0.3	15
Sorin	Bicarbon	11	1.5	23	1.9	34
	Allcarbon	3	1	2	1.2	5
Medtronic	OpenPivot	6	1.55	31	2.02	37
	Medtronic-Hall	0		11	1.3 ± 0.2	11
	Advantage	0		4	1.7 ± 0.2	4
Cryolife	On-X	0		2	1.7 ± 0.4	2
	Total	100		316		416

**Table 3:** Operative data of the matched sample.

Variable	PPM (*n* = 147)	No PPM (*n* = 147)	*p*
**Operative Data**			
Bypass time (mean ± SD)	108.05 ± 61.01	100.33 ± 38.06	0.19
Cross-clamp time (mean ± SD)	70.76 ± 25.52	67.37 ± 24.33	0.24
Postoperative length of stay (mean ± SD)	9.02 ± 4.54	9 ± 5.00	0.97
Early mortality (<30 days)	12 (8.16)	5 (3.40)	0.08
EOAi (cm^2^/m^2^)	0.71 ± 0.09	1.03 ± 0.17	<0.01
Euroscore 2 risk score	1.85 ± 1.97	1.74 ± 1.45	0.59
Follow-up (years) (mean ± SD)	6.54 ± 4.60	5.98 ± 4.47	0.32

**Table 4:** Average effects of PPM on the recipients.

_t	Coefficient	Robust SE	z	*p*	95% CI	
ATE (PPM vs. No PPM)	0.49	1.64	0.30	0.76	2.73	3.71
POmean (PPM vs. No PPM)	8.59	1.06	8.09	0.00	6.51	10.68

ATE = average treatment effects; POmean = potential outcome means.

**Table 5:** Average effect of severe PPM on long-term survival.

_t	Coefficient	Robust SE	z	*p*	95% CI	
ATE (Severe PPM vs. No severe PPM)	4.03	2.53	1.59	0.11	0.93	9.01
POmean (PPM vs. No PPM)	4.14	1.17	3.52	0.00	1.83	6.44

ATE = average treatment effects; POmean = potential outcome means.
